# Fascicle lengthening during a large torque reduction subsequently decreases dorsiflexion torque steadiness

**DOI:** 10.1038/s41598-026-52001-z

**Published:** 2026-05-26

**Authors:** Brent James Raiteri, Ricardo De Lorenzo, Malte Kraul, Daniel Hahn

**Affiliations:** 1 Human Movement Science, Faculty of Sport Science, Ruhr University Bochum, Bochum, North Rhine-Westphalia Germany; 2 School of Human Movement and Nutrition Sciences, The University of Queensland, Brisbane, Queensland Australia

**Keywords:** Activation reduction, Contraction history, Dorsiflexor, Force depression, Force enhancement, Isometric, Neurophysiology, Musculoskeletal system, Motor control

## Abstract

Here we assessed the effect of different rates and amplitudes of active torque reduction on the subsequent steady-state EMG amplitude, torque steadiness, and fascicle length during submaximal voluntary fixed-end (isometric) dorsiflexion contractions. Based on previous findings following active muscle-tendon unit lengthening, we expected a reduced steady-state EMG amplitude and torque steadiness relative to torque-matched reference contractions. In Experiment 1, thirteen participants performed reference contractions to 40% of maximum voluntary torque (MVT) and test contractions to 60 then 40% MVT over durations of 0.25, 0.5, 1 or 2 s. In Experiment 2, fourteen different participants performed reference contractions to 15, 30, and 45% MVT and test contractions to 85 then 15, 30, or 45% MVT at a fixed rate. Increasing the rate or amplitude of active torque reduction respectively increased tibialis anterior’s fascicle lengthening rate (1.6 to 6.9 mm·s^− 1^, *p*≤.041) or amplitude (3.6 to 7.5 mm; *p*<.001). However, fascicle lengthening did not subsequently change EMG amplitudes relative to the reference conditions (−1.5 to 2.7% MVC, *p*≥.102) at similar steady-state torques. Fascicle lengthening amplitudes of ≥ 8 ± 3% (5.2 ± 1.7 mm) reduced torque steadiness (0.7 to 1.5%, *p*≤.005) relative to reference conditions though, but the contributing mechanisms remain unclear and are worthy of further investigation.

**Supplementary Information:**

The online version contains supplementary material available at 10.1038/s41598-026-52001-z.

## Introduction

Skeletal muscles power and brake movement by generating force. Measuring internal muscle force directly is currently impossible and muscle force is conventionally estimated with non-trivial assumptions and estimates or measurements of net joint torque, which can be input into muscle-tendon models^1,2^. However, internal muscle force estimates are difficult because multiple muscles with changing moment arms contribute to the net joint torque^3^. Additionally, for a given activation, an individual muscle’s force depends on its instantaneous length^4^ and velocity^5^, as well as the muscle’s contraction history^6^. This contraction history is most obvious during continued maximal activation following active muscle shortening or lengthening when compared with an isometric (i.e. fixed end) reference contraction at the same final muscle length. Relative to this reference contraction, the steady-state muscle force remains depressed or enhanced following active muscle shortening or lengthening, respectively^6^. This so-called residual force depression (rFD) or enhancement (rFE) remains for as long as the muscle remains active^7^, thus complicating model-based predictions of muscle force.

Although some muscle models account for contraction history in their force predictions^8–10^, most muscle models do not^11–14^. Contraction history is often ignored to reduce model complexity, and because unbiased estimates of rFD or rFE are typically unavailable for a specific muscle in a given contraction. For example, what is often neglected is that the mechanisms underpinning rFD^15^ might depress force output during the fixed-end ‘reference’ contractions themselves. In support of this idea, recent findings indicate that increasing muscle fiber/fascicle shortening during active force production at a fixed muscle-tendon unit (MTU) length reduces steady-state force or torque output^16–18^. Ignoring this rFD in the fixed-end reference contractions can lead to rFD underestimation (3–4%) following MTU shortening^17^ and rFE overestimation (3–18%) following MTU lengthening^17,19^.

Only a few studies have attempted to quantify the effect of contraction history during fixed-end contractions^16–18^ and therefore representative findings following MTU lengthening will now be discussed as reflecting rFE. Following MTU lengthening, rFE is reflected by an enhanced steady-state torque output relative to that produced during electromyographic (EMG)-amplitude-matched, fixed-end reference contractions; rFE is most typically observed at long final muscle lengths on the descending limb of the muscle’s force-length relation^20^. Following MTU lengthening, rFE is also reflected by a reduced steady-state EMG amplitude (i.e. so-called ‘activation’ reduction) relative to torque-matched, fixed-end reference contractions^21,22^. There are also reports of reduced torque steadiness alongside ‘activation’ reduction following MTU lengthening^21,22^, with reduced torque steadiness being incorrectly attributed to less neural drive rather than increased variability in the common synaptic input. Reduced neural drive following active muscle lengthening can be mechanistically linked to rFE, whereby increased passive force contributions (e.g., from titin^23,24^ reduce the active force contribution and corresponding neural drive required to maintain a given joint torque^21^. However, what remains unclear is how the activation dynamics during fascicle lengthening affect these findings. Rather than inducing fascicle lengthening via MTU lengthening under constant activation, fascicle lengthening can also be induced during a contraction by a reduction in muscle activity (which is reflected by a decrease in EMG amplitude from surface EMG recordings), which causes in-series elastic tendon recoil and subsequent fascicle lengthening. Yet it remains unexplored if fascicle lengthening due to in-series elastic tissue recoil during a reduction in EMG amplitude can lead to rFE in terms of ‘activation’ reduction. Testing if a reduction in EMG amplitude triggers rFE-related mechanisms during fascicle lengthening is worthwhile because then an active torque reduction strategy could be used to decrease rFD contamination in fixed-end contractions.

Therefore, the purpose of this study was to determine the influence of the rate and amplitude of active torque reduction on muscle activity (not activation^25^ as reflected by EMG amplitude, torque steadiness, and fascicle length during submaximal voluntary fixed-end contractions. To induce fascicle lengthening, we instructed participants to reduce their activity level and torque output from a higher ‘test’ level to a lower ‘reference’ level within the same fixed-end contraction. The rate and amplitude of the active torque reduction was varied, with the aim of inducing in-series elastic tissue recoil of different rates and amplitudes, to subsequently lengthen tibialis anterior’s (TA’s) muscle fascicles at different rates and amplitudes. We expected that any fascicle lengthening would trigger rFE-based mechanisms within the fascicles that remained active during the so-called ‘test’ condition, enhancing their force output, leading to a lower steady-state EMG amplitude compared with a torque-matched reference condition with no preceding fascicle lengthening. We also expected, based on previous findings^21,22^, that fascicle lengthening during a reduction in EMG amplitude would lead to reductions in torque steadiness. However, in Experiment 1, we did not expect faster rates of fascicle lengthening to progressively reduce activity level because rFE is independent of lengthening speed at slow and moderate speeds^6,26,27^. On the contrary, in Experiment 2, we expected that increasing the fascicle lengthening amplitude would progressively increase the reduction in TA’s activity level because rFE is amplitude dependent over small to medium amplitudes^28,29^.

## Methods

### Participants

Fourteen and fifteen different participants, who were healthy (i.e. pain-, injury-, and fatigue-free) and recreationally-active, were recruited from the Faculty of Sport Science at Ruhr University Bochum to participate in one of two experiments (Experiment 1: age: 26 ± 3 year (mean ± standard deviation, SD); mass: 73 ± 14 kg; height: 1.76 ± 0.11 m, 6 women; Experiment 2: age: 26 ± 3 year, mass: 72 ± 9 kg, height: 1.76 ± 0.09 m, 5 women). All participants provided free written informed consent prior to participating in either experiment. Each experimental protocol was conducted in accordance with the Declaration of Helsinki and approved by the Ethics Committee of the Faculty of Sport Science at Ruhr University Bochum (Experiment 1: EKS V 33/2019; Experiment 2: EKS V 11/2021). G*Power (v3.9.1.7, RRID: SCR_013726^30^) determined that a minimum sample size of ten to thirteen was required to achieve at least 80 to 90% power to detect a minimum effect size of interest (*d*_z_) of 1 between two conditions of interest with a two-tailed alpha level of 5%. With these sample sizes, only large effects between two paired conditions could be detected, but large effects were expected (mean *d*_z_ = 1.39, range = 0.92 to 2.05) based on previous rFE findings in vivo from the human dorsiflexors^17,21,31–33^.

### Experimental setup

Participants were secured prone on a bench via a lashing strap with extended knees (similar to Fig. 1 of Rissmann et al.^34^) while they performed voluntary contractions with their right dorsiflexors against a motorized dynamometer. The participant’s shanks hung off the bench and the participant’s right foot was secured to the dynamometer’s footplate attachment with a custom-built adjustable U-shaped frame that was secured over the metatarsals, as well as a Velcro strap over the midfoot. This setup limited joint rotation during dorsiflexion contractions, as well as force contributions from the toe extensors to the measured net ankle joint torque. The heel of the participant’s right foot was also flush against the footplate’s rigid heel support, which prevented the foot from translating on the footplate, and foam was positioned between the back of the heel and footplate to minimize discomfort. The participant’s right ankle joint was fixed for the duration of the experiment at an angle of ~ 10° plantar flexion; 0° plantar flexion represents a perpendicular angle between the shank and sole (i.e. plantar aspect) of the foot. The tested angle was verified during a 50% perceived effort contraction with the help of a digital goniometer (EPT-DAF 380 mm, Conrad Electronic SE, Hirschau, Germany). Ankle joint misalignment was minimized during the 50% perceived effort contraction by manipulating the dynamometer’s axis of rotation to ensure that a laser pointer, which was projected along this axis, was located over the lateral malleolus of the right foot.

**Fig. 1:**
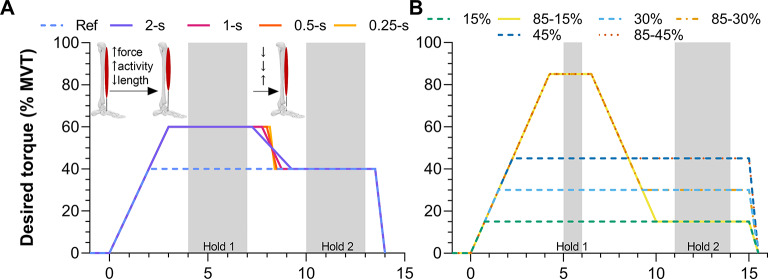
Desired normalized active torque traces that participants attempted to match during fixed-end dorsiflexion contractions in (**A**) Experiment 1 and (**B**) Experiment 2. All contractions lasted 13.5 s in Experiment 1 and 15 s in Experiment 2. The grey shaded areas indicate the two analyzed steady-state hold phases (Hold 1 and Hold 2). In Experiment 1, the ascending ramp rate was constant at 20% of maximum voluntary torque (MVT)·s^− 1^, whereas the descending ramp rate from Hold 1 to Hold 2 was variable. In Experiment 2, the ascending and descending ramp rates were constant at 20% MVT·s^− 1^. The absolute difference in normalized active torque between the measured and desired trace from contraction onset to the end of Hold 2 could not be more than 10% MVT at 10° plantar flexion for the trial to be considered valid.

The experimental techniques used are similar to those already described in Raiteri et al.^35^ and will only be briefly described here. The data collection system (Cambridge Electronic Design Ltd, Cambridge, UK) used a ± 5 V input range and a 16-bit analog-to-digital converter (Power1401-3) coupled with Spike2 software (64-bit version). This system temporally synchronized all recorded digital signals. Net ankle joint torque and crank arm angle were measured at 2 kHz via dynamometry (IsoMed2000, D&R Ferstl GmbH, Hemau, Germany). TA’s muscle activity level was based on its EMG amplitude assessed via a single differential signal recorded at 2 kHz via surface electromyography. Surface electrodes (hydrogel Ag/AgCl, 8 mm recording diameter, H124SG, Kendall International Inc, Mansfield, Massachusetts, United States) were attached to the skin above TA’s distal superficial compartment in a bipolar configuration (2 cm inter-electrode distance), as well as above the left lateral femoral condyle (reference electrode in Experiment 1) or right fibular head (reference electrode in Experiment 2), following standard skin preparation. TA muscle architecture changes were visualized via PC-based brightness-mode ultrasound imaging (LS128 CEXT-1Z, TELEMED, Vilnius, Lithuania) and a coupled flat, linear-array transducer (LV7.5/60/128Z-2). The transducer was secured over TA’s mid-belly just proximal to the bipolar electrodes with self-adhesive bandage. Ultrasound images were captured at ~ 34 fps with a frequency of 6 or 8 MHz and a 60 mm (width) × 50 mm (depth) field of view.

### Experimental protocol

Participants in either experiment performed the same respective protocol over two sessions. The familiarization session occurred on a separate day prior to the experimental session. The dorsiflexor MTUs were first preconditioned with five submaximal voluntary (80% perceived effort, 1-s hold, 1‐s rest) dorsiflexion contractions and one near-maximal (~ 95% perceived effort, 3-s hold) contraction^36^. After two minutes of rest, participants then performed two to four maximal voluntary contractions (MVCs; 3-s to 5-s hold) until the peak-to-peak torque was not different by more than 5% MVT between MVCs. Participants were told by the investigator to pull the top of their right foot towards their shank as hard as possible using only their dorsiflexor muscles and they received real-time visual feedback of their net ankle joint torque via a screen positioned in front of them. Participants rested their jaw in their hands so that they did not need to actively maintain neck extension to view the screen. At least two minutes of rest was provided to minimize fatigue following each MVC.

Once the highest MVT was determined, participants attempted to match their real-time active torque (i.e. baseline net joint torque at rest subtracted from the recorded net joint torque) during fixed-end dorsiflexion contractions to within predefined traces that were 6% MVT (Experiment 1) or 8% MVT (Experiment 2) apart. Torque was matched rather than activity level because torque variability during a contraction is lower and thus easier to match within predefined traces, especially during the dynamic phases of a contraction^16^. The mean desired torque traces for all conditions in both experiments are shown in Fig. 1; note that reference conditions with constant steady-state torque production (i.e. no torque drop) were included to ensure we could make time-matched comparisons to this baseline condition.

### Experiment 1

The ascending ramp was performed at 20% MVT/s to either 40% MVT in a reference condition, or to 60% MVT in four test conditions (Fig. 1A). After 60% MVT was achieved in the test conditions, the active torque was then reduced to 40% MVT as in the reference condition and then maintained during a second hold phase (Hold 2) that lasted between 4.25 s and 5.1 s. The duration of the descending ramps from 60 to 40% MVT varied between test conditions and were 0.25 s, 0.50 s, 1.00 s, or 2.00 s. Conditions were performed in blocks (i.e. five trials per block because there were five conditions), the order of conditions in each block was randomized, and at least four blocks (i.e. four contractions per condition) were performed. Within each block, a trial from the test condition was deemed invalid if the torque deviated 10% MVT or more from the desired torque trace, and invalid trials were immediately repeated up to three times. If the fourth consecutive trial was also deemed as invalid, then that condition was postponed and repeated after the fourth block was completed. At least one minute or two minutes of rest separated the trials and blocks, respectively.

### Experiment 2

The ascending and descending ramp rates were kept constant at 20% MVT/s among conditions in this experiment, but the ramp levels varied. The ascending ramp was either to 15% MVT, 30% MVT, or 45% MVT in three reference conditions, or to 85% MVT in three test conditions (Fig. 1B). A higher normalized torque than 85% MVT was not used because pilot testing revealed that this level was too high to consistently match for the duration of the experiment. After 85% MVT was achieved in the test conditions, the active torque was then reduced to 15% MVT (85 − 15% MVT), 30% MVT (85 − 30% MVT), or 45% MVT (85 − 45% MVT) as in each reference condition and then maintained (Hold 2) for 5 s, 5.75 s, and 6.5 s, respectively. The order of the other conditions in each block was randomized and at least three blocks (i.e. three contractions per condition) were performed. A trial from the test condition was deemed invalid if the torque deviated more than 10% MVT from the desired torque trace, and trials were repeated based on the same description as for Experiment 1. At least two or three minutes of rest separated the trials and blocks, respectively.

### Surface electromyography

Electromyography was performed using different systems in the two experiments (Experiment 1: AnEMG12, OT Bioelettronica, Torino, Italy; Experiment 2: NL 844 Pre-Amplifier and NeuroLog System, Digitimer Ltd, Hertfordshire, UK). Regardless of the system, EMG signals were amplified 1000 times and band-pass analog filtered between 10 and 500 Hz. Prior to electrode placement, ultrasound imaging was used to verify that the bipolar electrodes over the TA were in line with its muscle fascicles, as well as away from the muscle’s borders at rest and during contraction.

### Ultrasound imaging

A flat linear-array transducer was centered over the TA’s mid-belly in the sagittal plane in a custom-made polystyrene frame to minimize local muscle compression and discomfort. The transducer’s imaging face was coated in water-soluble transmission gel and secured over the skin once the fascicles and aponeuroses of TA’s superficial and deep compartments were clearly visible.

### Data analysis

Data were processed offline using custom-written scripts in MATLAB (R2022a 64-bit version, MathWorks, Natick, Massachusetts, United States, RRID: SCR_001622). First, the recorded digital signals from each trial were cropped between a common start and end time using the ultrasound timestamps and then exported as .mat files and combined with tracked fascicle data as previously described^17^. The tracked fascicle data included absolute lengths of a representative fascicle within TA’s superficial compartment, which was tracked automatically by applying the Lukas-Kanade-Tomasi algorithm using an updated version (https://github.com/brentrat/UltraTrack_v5_3) of UltraTrack^37^.

Dynamometer data were filtered using dual-pass second-order low-pass Butterworth filters with corrected cut-off frequencies^38^ of 20 Hz for net joint torque data and 6 Hz for crank arm angle data. Active torque was calculated by subtracting the filtered baseline torque from the filtered recorded torque of each trial. Baseline torque was calculated as the mean filtered recorded torque before contraction onset over 1 s (all submaximal voluntary contractions), 0.5 s (MVCs from Experiment 1), or 0.25 s (MVCs from Experiment 2). TA’s EMG amplitude was calculated as follows: first, the raw EMG signal was bandpass filtered between 20 and 400 Hz with a dual-pass second-order Butterworth filter; second, the mean bias was subtracted; third, this corresponding signal was rectified, and; lastly, the rectified signal was smoothed with a dual-pass second-order low-pass Butterworth filter with a corrected cut-off frequency^38^ of 10 Hz. Active torque and EMG amplitude data were then normalized to the corresponding values attained during the MVC trial (0.5-s window centered on the maximum amplitude) with the highest active torque (i.e. MVT).

Fascicle shortening amplitude was calculated as the maximum difference between fascicle lengths from 0.5 s before contraction onset until the start of Hold 2. Fascicle lengthening amplitude was calculated as the peak-to-peak difference in fascicle lengths between the end of Hold 1 and the start of Hold 2. Fascicle velocity was calculated as the maximum derivative of fascicle length over the same period. The mean values of active torque, EMG amplitude, and absolute fascicle length during Hold 1 and Hold 2 were quantified for each trial of each condition for both experiments. These three outcome variables, as well as fascicle shortening and lengthening amplitudes, the maximum fascicle lengthening speed, the maximum torque matching error (see paragraph below), and torque steadiness (see next sentence) were then averaged among all valid trials within each condition and statistically analyzed. Torque steadiness was calculated as the coefficient of variation (CV) in active torque during Hold 2.

The ability of the participants to match the desired torque trace was assessed by subtracting the active torque from the mean of the two displayed torque traces over a period from contraction onset until the end of Hold 2 (Fig. 1). The ascending ramp was included in this calculation so that fascicle shortening velocities and motor unit discharge rates would likely be similar among conditions^39^. The maximum deviation in torque matching was then normalized to the MVT. Trials with torque matching errors of more than 10% MVT were excluded from analysis. Valid trials were also required to have active torques between the end of Hold 1 and start of Hold 2 that were not more than 0.8 Nm (Experiment 1) or 2 Nm (Experiment 2) below the mean active torque during Hold 2. Values of 0.8 Nm and 2 Nm were chosen because pilot testing revealed that the mean ±﻿ SD torque variability among conditions during Hold 2 was 0.6 ± 0.1 Nm (Experiment 1) and 0.8 ± 0.6 Nm (Experiment 2), and we opted for a threshold that was two standard deviations above these mean torque variabilities.

### Statistics

Statistical analysis was performed in GraphPad Prism (9.1.2 64-bit version, San Diego, California, United States, RRID: SCR_002798) with a (two-tailed) 5% alpha level. A two-way repeated-measures mixed-effects analysis was performed to identify mean differences among conditions for the independent variable, normalized active torque (% MVT). One-way repeated-measures mixed-effects analyses were performed to identify mean differences among conditions during Hold 2 in torque matching error, normalized EMG amplitude (% MVC), absolute fascicle length, and torque steadiness. The same analyses were performed for fascicle lengthening amplitude and maximum fascicle lengthening speed. The Greenhouse Geisser correction was applied when sphericity was violated based on data from or before Hold 2 as assessed via Mauchly’s test in MATLAB. Following a significant effect or interaction, Holm-Sidak multiple comparisons were performed among conditions; a single pooled variance was used when sphericity was not violated, otherwise individual variances were used. Lastly, repeated-measures correlations were calculated to assess the strength of the linear relation between: (1) active torque and EMG amplitude during Hold 1 and 2 in the test conditions of each experiment; (2) torque matching error and torque steadiness (i.e. CV in active torque) during Hold 2 in the test conditions of Experiment 2, and; (3) torque steadiness and fascicle lengthening amplitude in the test conditions of each experiment. Data are presented as mean ± SD.

## Results

### Experiment 1: active torque reductions at different rates

#### Data exclusion

Results from Experiment 1 are based on *n* = 13 (group age: 26 ± 2 year; mass: 71 ± 13 kg; height: 1.75 ± 0.11 m; 6 women, age: 25 ± 1 year; mass: 62 ± 4 kg; height: 1.69 ± 0.05 m; 7 men, age: 27 ± 3 year; mass: 81 ± 13 kg; height: 1.82 ± 0.10 m). One of the fourteen tested participants had no valid trials in the 0.5-s condition and their dataset was excluded from statistical analysis. Fascicle kinematics from two additional participants were excluded because of inaccurate automated fascicle tracking based on visual inspection; resulting in *n* = 11 for fascicle kinematic data.

#### Torque matching error

A total of 22 ± 4 (range: 19–34) submaximal voluntary contractions per participant were recorded and 16 ± 2 (range: 13–21) were valid. Per test condition (see Fig. 1A), 2 ± 1 (0.25-s), 3 ± 1 (0.5-s), and 4 ± 1 trials (1-s, 2-s) were valid, and 4 ± 1 trials were valid in the reference condition. The maximum active dorsiflexion torque at 10 ± 1° plantar flexion was 40.5 ± 9.0 Nm (range: 30.4-62.4 Nm). The torque matching error relative to the desired trace from contraction onset until the end of Hold 2 (Fig. 1A) was different among conditions (*F*_2.42,29.09_=19.96, *p*<.001), with higher torque matching error in the test conditions (0.25-s: 7.6 ± 1.1%; 0.5-s: 7.0 ± 1.0%; 1-s: 6.4 ± 1.0%; 2-s: 6.7 ± 1.0% MVT) versus reference condition (5.3 ± 0.8% MVT, *p*<.001).

#### Hold 1

**Fig. 2:**
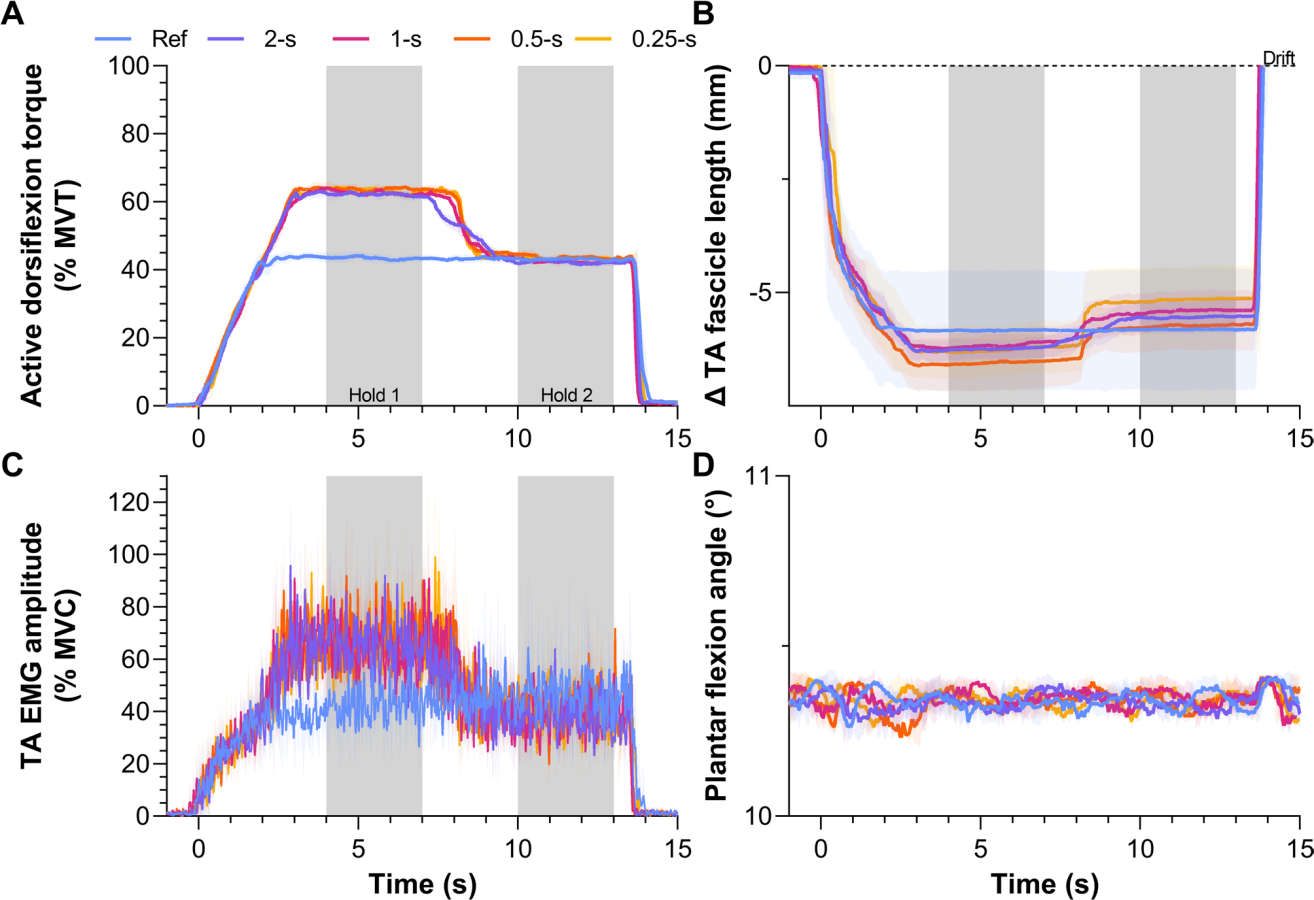
Mean and standard deviation (shaded areas) (**A**) normalized active torque-time, (**B**) tibialis anterior (TA) muscle fascicle length change-time, (**C**) normalized TA EMG amplitude-time, and (**D**) crank arm angle-time traces for a representative participant in Experiment 1. The grey shaded areas indicate the two analyzed steady-state phases (4–7 s and 10–13 s) of the contractions. The conditions included one reference condition at 40% maximum voluntary torque (MVT) and four test conditions at 60% MVT then 40% MVT. The test conditions differed in the descending ramp duration from Hold 1 to Hold 2, which was either over 0.25, 0.5, 1 or 2 s. The test conditions thus differed in the rate of active torque reduction. In B, fascicle length changes are relative to the passive fascicle length prior to contraction onset; the y axis has been cut at zero because fascicle lengths drifted beyond their passive length prior to contraction onset following the contraction. In C, EMG amplitudes are based on full-wave rectified surface EMG signals that were smoothed with a second-order zero-lag Butterworth filter at 10 Hz and then normalized to maximal voluntary contraction.

As intended, the steady-state active torque (Fig. 2A) during Hold 1 was different among conditions (*F*_2.16,25.91_=683.70, *p*<.001), and there was an interaction between condition and hold phase (*F*_2.55,30.57_=4619, *p*<.001). Active torque was higher in the test conditions compared with the reference condition (∆19.9 ± 0.6 to ∆20.0 ± 1.4% MVT, *p*<.001), but similar among test conditions (∆0.0 ± 1.2 to ∆0.1 ± 0.4% MVT, *p*≥.983; Table 1).

**Table 1:** Normalized active torques during Hold 1 and 2, tibialis anterior (TA) fascicle lengthening amplitudes and rates from Hold 1 to 2, and normalized TA EMG amplitudes and absolute fascicle lengths during Hold 2 among the five fixed-end conditions in Experiment 1.

	Hold 1	Hold 1 to Hold 2		Hold 2		
	Torque (% MVT)	Lengthening amp. (mm)^§^	Lengthening rate (mm·s^− 1^)^§^	Torque (% MVT)	EMG amp. (% MVC)	Length (mm)^§^
Reference	40.2 (1.3)^*^	0.1 (0.1)^*^	0.6 (0.2)^*^	40.0 (1.3)	35.7 (11.9)	83.3 (11.7)
2-s	60.0 (1.1)	0.5 (0.2)^*^	1.6 (0.8)^*^	40.7 (1.0)	34.1 (8.9)	83.0 (11.8)
1-s	60.1 (1.2)	0.5 (0.2)^*^	2.1 (1.0)^*^	40.6 (1.1)	34.8 (9.9)	82.9 (11.4)
0.5-s	60.1 (1.7)	0.7 (0.4)^*^	3.6 (2.6)^*^	40.6 (1.4)	35.2 (10.1)	82.9 (11.9)
0.25-s	60.1 (2.0)	0.9 (0.4)^*^	6.9 (4.1)^*^	40.6 (2.0)	35.7 (10.9)	83.2 (11.4)

Values are means (standard deviations); *n* = 11^§^/13. MVT, maximum voluntary torque; amp., amplitude; MVC, maximal voluntary contraction. ^*^Different to other conditions (*p* < .05) based on a two-way repeated-measures mixed-effects model with Holm-Sidak post-hoc corrections.

#### Descending ramp

**Fig. 3:**
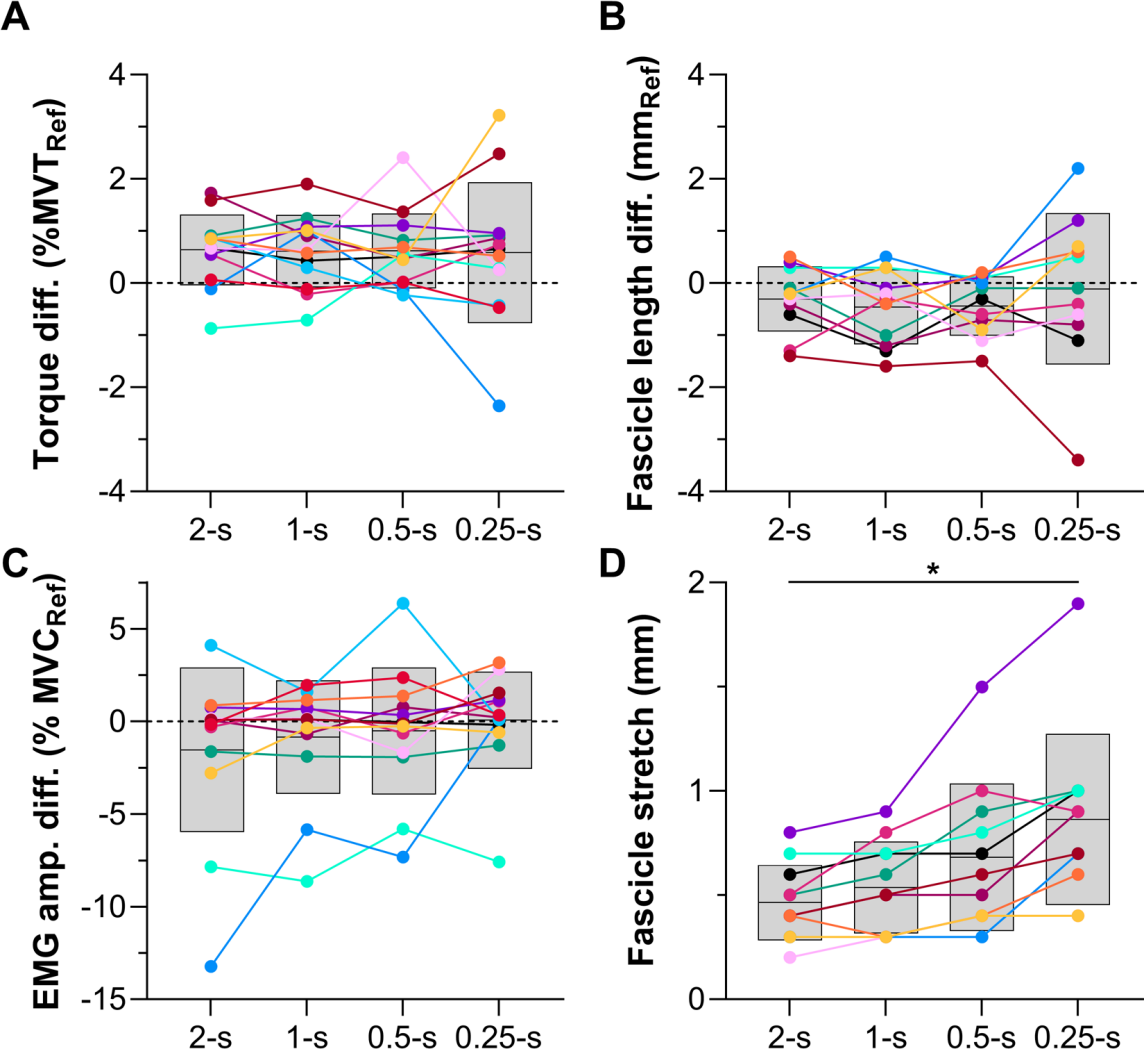
Individual and mean (black horizontal lines) differences for (**A**) normalized active torque, (**B**) tibialis anterior (TA) fascicle length, and (**C**) normalized TA EMG amplitude during Hold 2 of the four test conditions relative to the reference condition in Experiment 1. Individual and mean differences for (**D**) TA fascicle lengthening amplitude from Hold 1 to Hold 2 are also shown. Each color represents a different participant and grey shaded areas indicate standard deviations. Two-way (active torque only) or one-way repeated-measures mixed-effects analyses with a Greenhouse-Geisser correction and post-hoc individual-variance-based Holm-Sidak multiple comparisons were performed to identify mean differences among conditions. The asterisk in D indicates that the fascicle lengthening amplitudes were different among all conditions.

The fascicle lengthening amplitudes (Table 1; Fig. 3D) and rates from the end of Hold 1 to the start of Hold 2 (Fig. 1A) were different among conditions (amplitude: *F*_1.51,15.13_=31.77, *p*<.001; rate: *F*_1.19,11.93_=20.81, *p*<.001). As intended, lengthening amplitudes and rates were higher in the test conditions compared with the reference condition (∆0.4 ± 0.2 to ∆0.8 ± 0.4 mm, *p*≤.001; ∆0.9 ± 0.6 to ∆6.2 ± 4.0 mm·s^− 1^, *p*≤.011; Table 1). Unexpectedly, the fascicle lengthening amplitudes were also different among all test conditions (range: 0.2–1.9 mm [0.3-2.0% of the length before lengthening]), but these differences were smaller (∆0.1 ± 0.1 to ∆0.4 ± 0.3 mm, *p*≤.040; Table 1) than those between the test and reference conditions. As designed, the fascicle lengthening rates were different among all test conditions (range: 0.6–16.7 mm·s^− 1^), which indicates that the different torque-drop rates effectively modulated the rate of fascicle lengthening (∆0.6 ± 0.6 to ∆5.3 ± 3.6 mm·s^− 1^, *p*≤.041; Table 1).

#### Hold 2

As torque was matched during Hold 2 among the test and reference conditions, the active torques among all conditions were similar (∆-0.1 ± 1.2 to ∆0.6 ± 1.4% MVT, *p*≥.051; Table 1; Fig. 3A), as were the fascicle lengths (*F*_1.71,17.05_=1.13, *p*=.337; ∆-0.5 ± 0.7 to ∆-0.1 ± 1.5 mm; Fig. 3B; Table 1). However, contrary to our expectations, the mean rectified EMG amplitudes during Hold 2 were not significantly different among the test and reference conditions (*F*_1.93,23.16_=1.24, *p*=.307; ∆-1.5 ± 4.4 to 0.1 ± 2.6% MVC; Table 1; Fig. 3C). Torque steadiness during Hold 2 was different among conditions (*F*_4,48_ =2.94, *p*=.030), being lower as reflected by a higher CV in active torque in the 0.25-s condition (1.7 ± 0.3%, *p*=.038) compared with the reference condition (1.4 ± 0.3%). However, this was a relatively small difference, and there were no other significant differences in torque steadiness among conditions (∆0.0 ± 0.3 to ∆0.2 ± 0.3%, *p* ≥ .094).

#### Hold 1 vs. Hold 2

The repeated-measures correlation between active torque and TA’s EMG amplitude in the test conditions was positive, strong and significant (*r*_rm_(90) = 0.91 [95% CI: 0.86 to 0.94], *p* < .001). However, the repeated-measures correlation between torque steadiness and TA’s fascicle lengthening amplitude in the test conditions was small and not significant (*r*_rm_(32) = 0.27 [95% CI: -0.07 to 0.56], *p* = .117).

### Experiment 2: active torque reductions of different amounts

#### Data exclusion

Results from Experiment 2 are based on *n* = 14 (group age: 26 ± 3 year; mass: 71 ± 9 kg; height: 1.77 ± 0.09 m; 5 women, age: 25 ± 1 year; mass: 62 ± 3 kg; height: 1.67 ± 0.05 m; 9 men, age: 26 ± 4 year; mass: 76 ± 6 kg; height: 1.82 ± 0.06 m). One of the fifteen tested participants was to unable to match the desired torque reduction traces in any test condition and their dataset was excluded from statistical analysis. Fascicle kinematics from two additional participants were excluded either because their ultrasound images and analog data could not be temporally synchronized or because of inaccurate automated fascicle tracking based on visual inspection; resulting in *n* = 12 for fascicle kinematic data.

#### Torque matching error

A total of 22 ± 2 (range: 18–25) submaximal voluntary contractions per participant were recorded and 17 ± 3 (range: 11–23) were valid. Per test condition (see Fig. 1B), 2 ± 2 (85 − 15% MVT), 2 ± 1 (85 − 30% MVT), and 3 ± 1 trials (85 − 45% MVT) were valid. Per reference condition, 3 ± 1 (15% MVT), 4 ± 1 (30% MVT), and 3 ± 1 trials (45% MVT) were valid. The maximum active dorsiflexion torque at 10 ± 0° plantar flexion was 39.2 ± 10.2 Nm (range: 27.8-54.9 Nm). The torque matching error relative to the desired trace from contraction onset until the end of Hold 2 (Fig. 1B) was different among conditions (*F*_5,65_=59.35, *p*<.001), with greater torque matching error during the test conditions (85 − 15/85 − 30/85 − 45% MVT: 8.5 ± 1.0/8.4 ± 1.2/8.4 ± 1.1% MVT) relative to each respective reference condition (15/30/45% MVT: 3.8 ± 0.6/4.9 ± 1.6/5.6 ± 1.1% MVT, *p*<.001).

#### Hold 1

**Fig. 4:**
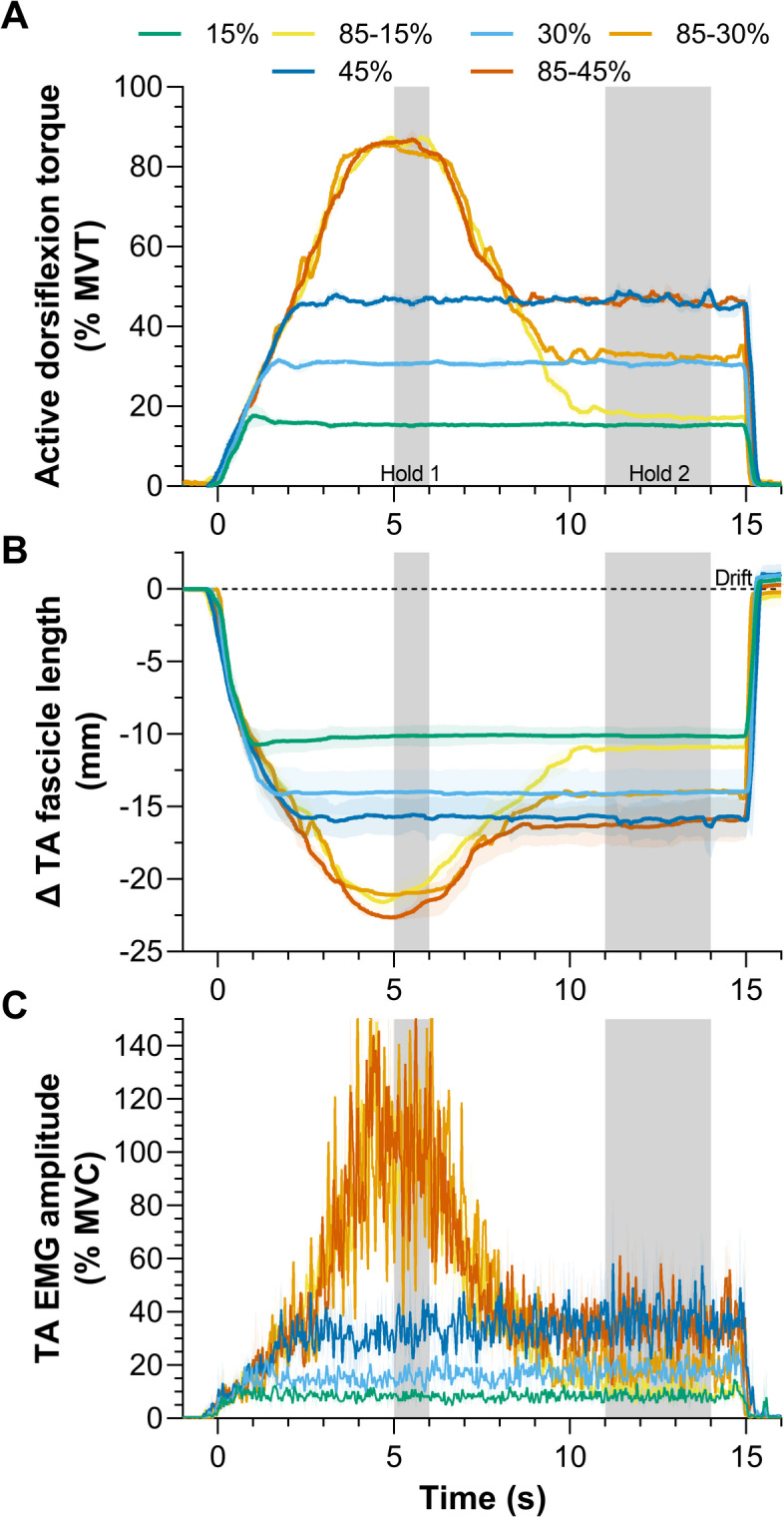
Mean and standard deviation (shaded areas) (**A**) normalized active torque-time, (**B**) tibialis anterior (TA) muscle fascicle length change-time, and (**C**) normalized TA EMG amplitude-time traces for a representative participant in Experiment 2. The grey shaded areas indicate the two analyzed steady-state phases (5–6 s and 11–14 s) of the contractions. The conditions included three reference conditions at 15% MVT, 30% MVT, and 45% MVT and three test conditions at 85% MVT then matched torque levels (i.e. 85 to 15% MVT, 85 to 30% MVT, and 85 to 45% MVT). The test conditions thus differed in the amount of active torque reduction. In B, fascicle length changes are relative to the passive fascicle length prior to contraction onset and optical-flow-based tracking drift is evident as the resting fascicle lengths following the contraction deviate from zero. In C, EMG amplitudes are based on full-wave rectified EMG signals that were smoothed with a second-order zero-lag Butterworth filter at 10 Hz and then normalized to maximal voluntary contraction. Note that in C, the y axis has been cropped from 202% MVC to 150% MVC to improve visibility of the mean traces.

As intended, the steady-state active torque (Fig. 4A) during Hold 1 was different among conditions (*F*_5,65_ =5366, *p*<.001), and there was an interaction between condition and hold phase (*F*_5,65_=4728, *p*<.001). Active torque was higher in each test condition compared with the respective reference condition (range of mean differences: ∆38.3 ± 1.8 to ∆67.9 ± 1.8% MVT, *p*<.001; Table 2). Additionally, active torque was different among reference conditions (∆14.7 ± 1.8 to ∆30.0 ± 1.8% MVT, *p*<.001), but similar among test conditions (∆0.0 ± 1.8 to ∆0.3 ± 1.8% MVT, *p*≥.855; Table 2).

**Table 2:** Normalized active torques during Hold 1 and 2, tibialis anterior (TA) fascicle lengthening amplitudes and rates from Hold 1 to 2, and normalized TA EMG amplitudes and absolute fascicle lengths during Hold 2 among the six fixed-end conditions in Experiment 2.

	Hold 1	Hold 1 to Hold 2		Contraction onset to Hold 2		
	Torque (% MVT)	Lengthening amp. (mm)^§^	Lengthening rate (mm·s^− 1^)^§^	Torque (% MVT)	EMG amp. (% MVC)	Length (mm)^§^
15% MVT	15.6 (1.1)^*^	0.01 (0.1)^*^	0.7 (0.3)^*^	15.4 (1.0)	11.9 (3.2)	79.4 (13.8) ^*^
85 − 15% MVT	83.5 (3.2)	7.5 (2.4)	8.3 (4.2)	16.1 (0.9)	11.7 (2.4)	78.1 (13.9)
30% MVT	30.3 (1.3)^*^	0.1 (0.1)^*^	1.0 (0.5)^*^	30.4 (1.1)	20.2 (3.5)	76.8 (13.7)^*^
85 − 30% MVT	83.5 (3.4)	5.2 (1.7)	7.3 (3.1)	31.5 (1.3)	20.4 (3.6)	75.3 (13.6)
45% MVT	45.6 (0.7)^*^	0.3 (0.2)^*^	1.8 (1.1)^*^	45.6 (1.2)	34.9 (6.4)	74.5 (13.8)^*^
85 − 45% MVT	83.8 (3.0)	3.6 (1.0)	6.7 (2.1)	45.9 (1.3)	32.2 (5.6)	73.7 (13.5)

Values are means (standard deviations); *n* = 12^§^/14. MVT, maximum voluntary torque; amp., amplitude; MVC, maximal voluntary contraction. ^*^Different to respective test condition (*p* ≤ .05) based on a two-way (torque) or one-way (length) repeated-measures mixed-effects model with Holm-Sidak post-hoc corrections.

#### Descending ramp

**Fig. 5:**
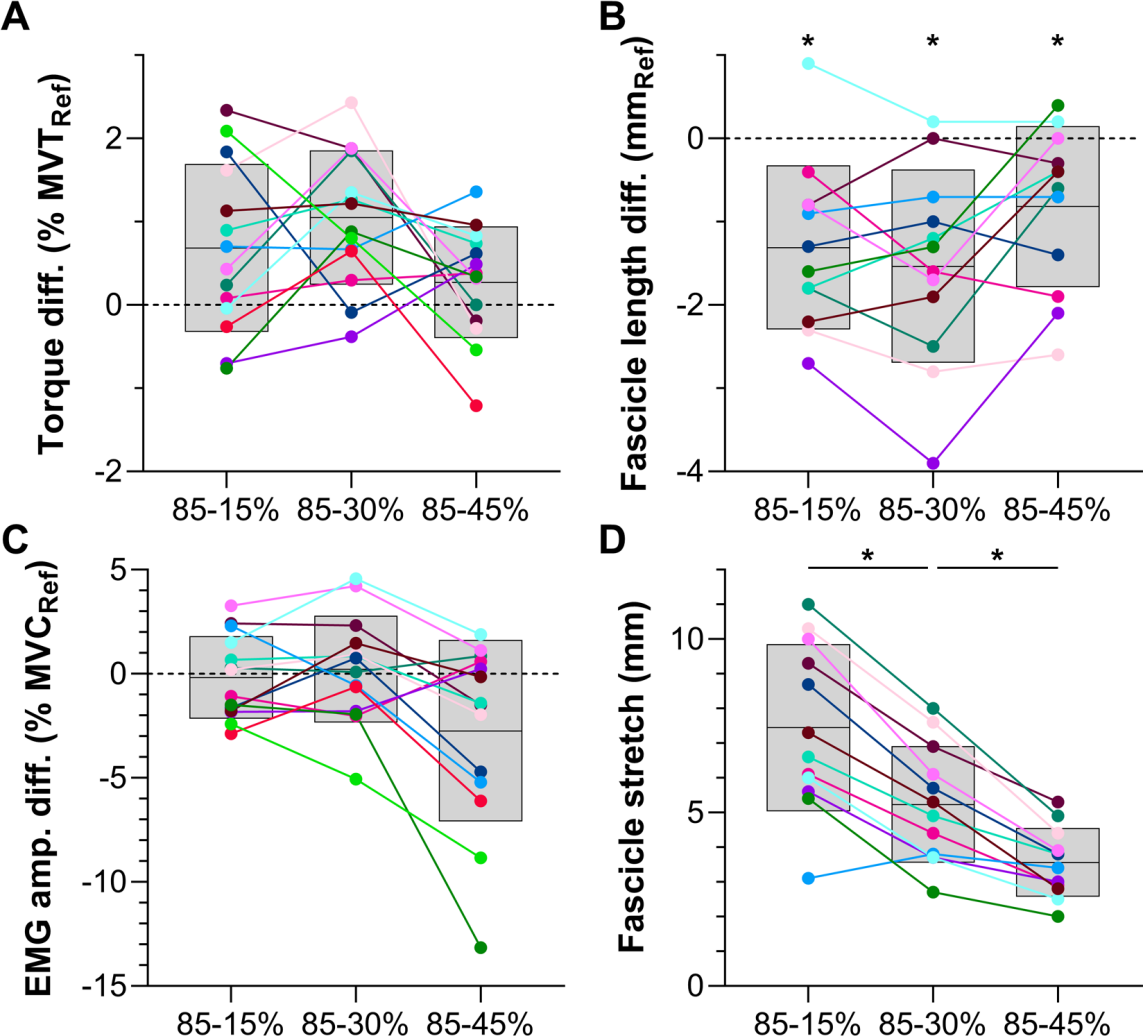
Individual and mean (black horizontal lines) differences for (**A**) active torque, (**B**) tibialis anterior (TA) fascicle length, and (**C**) TA EMG amplitude during Hold 2 of the three test conditions relative to the respective reference condition in Experiment 2. Individual and mean differences for (**D**) TA fascicle lengthening amplitude from Hold 1 to Hold 2 are also shown. Each color represents a different participant and grey shaded areas indicate standard deviations. Two-way (active torque only) or one-way repeated-measures mixed-effects analyses with a Greenhouse-Geisser correction and post-hoc individual-variance-based Holm-Sidak multiple comparisons were performed to identify mean differences among conditions. The asterisks in B indicate that mean fascicle lengths were shorter in the test conditions than the respective reference conditions, and the asterisks in D indicate that mean fascicle lengthening amplitudes progressively decreased from 85 − 15 to 85 − 30 to 85 − 45% MVT.

The fascicle lengthening amplitudes (Table 2; Fig. 5D) and rates from the end of Hold 1 to the start of Hold 2 (Fig. 1B) were different among conditions (amplitude: *F*_1.26,13.88_=109.2, *p*<.001; rate: *F*_1.90,20.92_=43.74, *p*<.001). As intended, lengthening amplitudes and rates were higher in the test conditions compared with the respective reference condition (∆3.3 ± 0.9 to ∆7.3 ± 2.4 mm, *p*<.001; ∆5.0 ± 1.7 to 7.6 ± 4.1 mm·s^− 1^, *p*<.006; Table 2). Additionally, among test conditions, the fascicle lengthening amplitudes (range: 2.0–11.0 mm [3.3–22.3%]) were different (∆1.7 ± 0.9 to ∆3.9 ± 1.8 mm, *p*<.001) while the fascicle lengthening rates were similar (∆0.5 ± 1.4 to ∆1.6 ± 3.3 mm·s^− 1^, *p*≥.344; Table 2), as intended. Consequently, different torque-drop amplitudes effectively modulated the amount, but not rate, of fascicle lengthening.

#### Hold 2

**Fig. 6:**
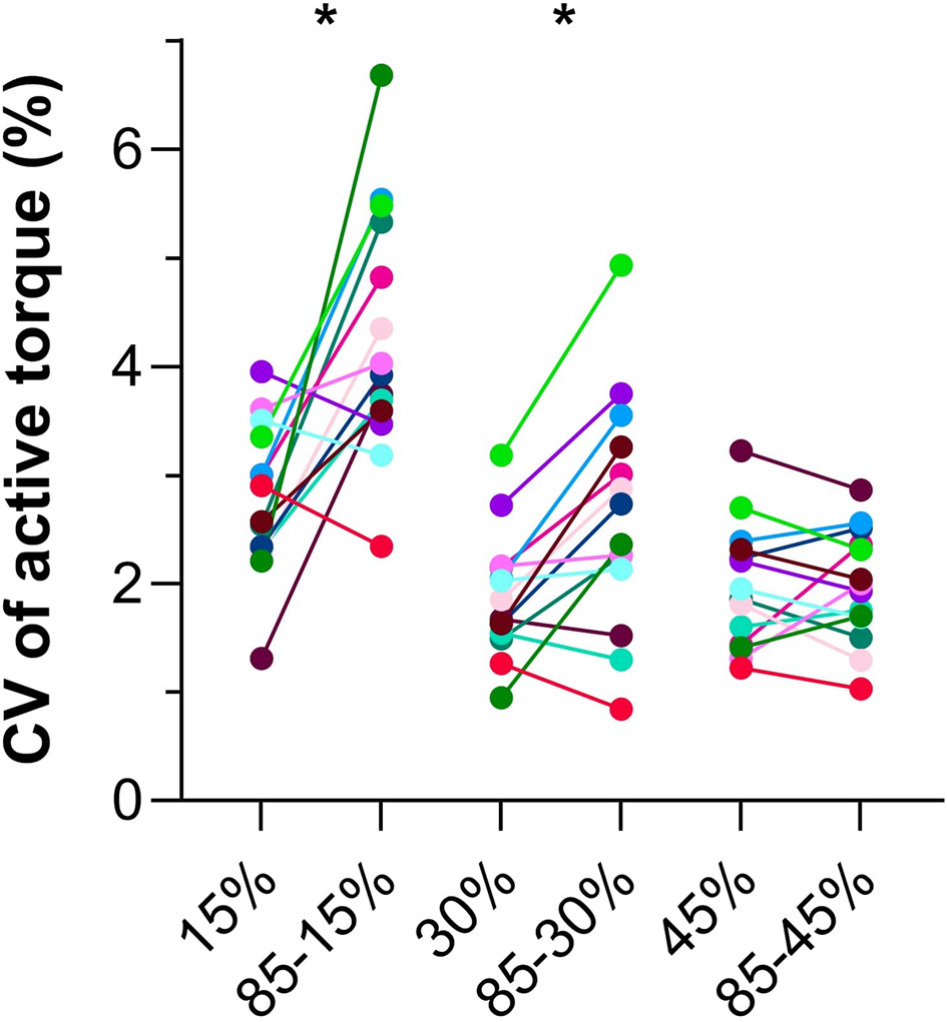
The coefficient of variation (CV) in active torque during Hold 2 in Experiment 2. Each color represents a different participant. A one-way repeated-measures mixed-effects analysis with a Greenhouse-Geisser correction and post-hoc individual-variance-based Holm-Sidak multiple comparisons were performed to identify mean differences among paired conditions. An asterisk indicates that the mean CV was higher in the test condition than the respective reference condition.

As torque was matched during Hold 2 among paired test and reference conditions, the active torques were similar (∆0.3 ± 1.8 to 1.1 ± 1.8% MVT, *p*≥.078; Fig. 5A). However, the fascicle lengths during Hold 2 were different between each paired test and reference condition (*F*_2.37,26.02_=76.22, *p*<.001; ∆-0.8 ± 1.0 to ∆-1.5 ± 1.2 mm, *p*≤.014; Fig. 5B; Table 2), which was not expected, but might be explained by fascicle tracking drift differences between the test and reference conditions (Fig. 4B). Although the mean rectified EMG amplitudes during Hold 2 were different among test conditions or reference conditions (*F*_2.26,29.37_=183.3, *p*<.001), again, contrary to our expectations, EMG amplitudes were not significantly different between each paired test and reference condition (∆-0.2 ± 2.6 to 2.7 ± 4.4% MVC, *p*≥.102; Fig. 5C). In line with previous findings, torque steadiness during Hold 2 (Fig. 6) was different among conditions (*F*_2.82,36.60_=24.38, *p*<.001), being lower as reflected by a higher CV in active torque between the 85 − 15% MVT condition (4.3 ± 1.1%, *p*=.004) and 15% MVT condition (2.8 ± 0.7%), as well as the 85 − 30% MVT condition (2.6 ± 1.1%, *p*=.005) and 30% MVT condition (1.9 ± 0.6%). However, torque steadiness was similar between the 85 − 45% MVT condition (2.0 ± 0.5%, *p*=.938) and 45% MVT condition (2.0 ± 0.6%).

#### Hold 1 vs. Hold 2

The repeated-measures correlation between active torque and TA’s EMG amplitude was positive, strong and significant (*r*_rm_(69) = 0.98 [95% CI: 0.96 to 0.99], *p* < .001), and this relation was likely stronger than that observed in Experiment 1 because of the greater range of active torques produced. There was no significant repeated-measures correlation between the torque matching error and torque steadiness in the test conditions (*r*_rm_(27)=-0.01 [95% CI: -0.37 to 0.36], *p* = .974). The repeated-measures correlation between torque steadiness and TA’s fascicle lengthening amplitude in the test conditions was positive, strong and significant (*r*_rm_(23) = 0.72 [95% CI: 0.46 to 0.87], *p* < .001).

## Discussion

In this study, we assessed if fascicle lengthening at a fixed MTU length during a reduction in muscle activity based on TA’s EMG amplitude could lead to a subsequently lower EMG amplitude or reduced torque steadiness relative to reference conditions at similar steady-state net joint torques. The test conditions involved initially higher torques and a subsequent reduction in active torque to a lower reference torque to induce fascicle lengthening via in-series elastic tissue recoil. The reference conditions involved constant torque production without preceding fascicle lengthening. Contrary to our expectations, we found that fascicle lengthening of different rates (Experiment 1) or amplitudes (Experiment 2) did not lead to a lower EMG amplitude relative to the reference conditions. However, partly in line with our expectations, fascicle lengthening of 8% or more (of the length before lengthening) and correspondingly large torque reductions (55 to 70% MVT) reduced torque steadiness by 0.7 to 1.5% relative to the reference conditions. Consequently, different histories of muscle length change subsequently affected torque steadiness when the lengthening amplitude was large enough, without significantly altering TA’s EMG amplitude at a given net joint torque. As such, it appears that the rFD present following fascicle shortening at a given muscle length cannot be eliminated by mechanisms triggered during fascicle lengthening and EMG amplitude reduction. Therefore, reducing the active torque applied is not an effective strategy to decrease rFD contamination in fixed-end contractions.

This study is different to most previous studies that investigated contraction history effects because we did not lengthen the MTU under constant activation to induce fascicle lengthening, but we induced fascicle lengthening via in-series elastic tissue recoil during a reduction in active torque at a fixed MTU length. In Experiment 1, we found that different active torque reduction rates effectively altered TA’s fascicle lengthening rate. However, we did not expect these differences in fascicle lengthening rate to progressively lower TA’s steady-state EMG amplitude relative to a torque-matched reference condition. We did not expect progressively lower EMG amplitudes following increased rates of fascicle lengthening because previous findings from the cat soleus and the human adductor pollicis muscles showed that rFE was unaffected by slow to moderate lengthening rates (2 to 32 mm/s^27^; 10 to 60°/s^40^). But, unlike these former studies, we did not find any indirect evidence of rFE as steady-state EMG amplitudes were not significantly lower following fascicle lengthening relative to the reference conditions. The only difference we observed was reduced torque steadiness following the fastest fascicle lengthening rate compared with the reference condition. But this difference was small (0.3 ± 0.3%) and may not be meaningful, especially considering that this condition had the highest torque matching error and the fewest valid trials between participants.

It is possible that the fascicle lengthening amplitudes were too small in Experiment 1 (i.e. a maximum of 2 mm or 2%) to trigger rFE-based mechanisms. This speculation is supported by our previous work, where we found no indirect evidence of rFE based on similar steady-state torques (2 ± 5%) between test and EMG-amplitude-matched reference contractions with (4 ± 2 mm or 4%) or without respective fascicle lengthening of the human vastus lateralis^20^. Alternatively, the amplitudes of shortening (7%) relative to lengthening (1%) in Experiment 1 might have been too great for the subsequent lengthening-induced rFE to overcome the previous shortening-induced rFD. This speculation is supported by previous findings from the cat soleus muscle of similar steady-state forces during fixed-end reference contractions versus following MTU shortening-lengthening cycles with 50% more shortening than lengthening^41^. Although both previous studies used MTU lengthening under a constant activity/activation level to induce fascicle lengthening, which is not directly comparable to the current study, their findings support a lack of activity level reduction in Experiment 1. Unfortunately though, we were not able to look at the effect of fascicle lengthening rate over larger lengthening amplitudes in this experiment because of the difficulty of reducing active torque at fast rates over amplitudes larger than 20% MVT.

In Experiment 2, fascicle lengthening amplitudes were larger (i.e. a maximum of 11 mm or 22%) and likely sufficient to induce rFE based on previous findings^29^. However, again we found no activity level reduction in the test versus reference conditions. Once more, it is possible that rFD masked rFE^41^ because the amplitudes of shortening (15%) exceeded lengthening (8%) by almost 50%. However, we have observed rFE before (11 ± 6%) when fascicle shortening exceeded lengthening by ~ 75% at relatively long vastus lateralis fascicle lengths on the descending limb of its force-length relation^42^, so the amount that rFD masks rFE is probably muscle-length dependent^20^. Consequently, rFE might have been absent in our experiments because we tested around the plateau of TA’s force-length relation^35^. However, this neglects our previous finding of rFE or reduced rFD (3–5%) within the same muscle group and region of the force-length relation^17^. Therefore, it is more likely that the rFE induced by fascicle lengthening during a reduction in EMG amplitude was insufficient to overcome the initial rFD induced by fascicle shortening from active force development.

One key difference between Experiment 1 and 2 that we did not expect and remains unexplained is related to torque steadiness. Torque steadiness as assessed by the CV in active torque was 0.7 to 1.5% more variable following fascicle lengthening amplitudes of 5 ± 2 mm (8 ± 3%) and 7 ± 2 mm (11 ± 5%) in Experiment 2. However, whether this finding can be mechanistically explained by rFE-based mechanisms, such as increased titin forces^23,24^, is unlikely because reduced torque steadiness was not accompanied by an EMG amplitude reduction, unlike in previous studies on the human dorsiflexors that compared MTU-lengthening-hold and fixed-end reference conditions^21,22^. Consequently, constant or increasing activation during fascicle lengthening might be required for subsequent EMG amplitude reduction in vivo, and the reduced torque steadiness following large EMG amplitude reductions in Experiment 2 is unlikely to be explained by rFE. Instead, there might be neural mechanisms at play that reduce torque steadiness following large EMG amplitude reductions, such as prolonged motor unit firing from persistent inward currents (PICs)^43^. Such a mechanism could certainly explain our torque steadiness and activity level findings, considering that increased torque variability has recently been associated with more active motor units (i.e. higher-threshold units) firing at lower average rates during fixed-end ‘sombrero’ contractions^43^. Although this interpretation cannot be explored further with our data as we did not use high-density EMG, future experiments could gain further insight into how PICs or rFE contribute to torque steadiness by performing similar contraction conditions to our Experiment 2, but at short (i.e. increasing PICs) or long (i.e. increasing rFE) muscle lengths^20,43^.

Aside from PICs, previous studies suggest that torque fluctuations are better explained by variability in the neural drive to the muscle rather than the variability in individual motor unit discharge times at moderate contraction intensities (5–60% MVT) of the TA^44^ and at moderate (20% MVT) but not low (5% MVT) contraction intensities of the first dorsal interosseus muscle^45^. Consequently, we speculate that greater modulations in the common synaptic input following large reductions (55–70% MVT) in active torque might help to explain the impaired torque steadiness we and others^43,46^ have observed, rather than increased variability in individual synaptic input or differences in intrinsic muscle properties. However, although the idea that greater neural drive modulations impair torque steadiness is in line previous interpretations^44,47,48^, more work needs to be done to understand why neural drive is less stable following fascicle lengthening and a reduction in EMG amplitude. Further work is also required to identify if neural drive is even less stable during similar contraction conditions in individuals with neuromuscular disorders, and if so, it might be worth using the test conditions from Experiment 2 alongside high-density EMG measurements to attempt to detect disease status and monitor progression.

It is important to remember that this study does not provide conclusive evidence that rFE is not induced by fascicle lengthening during a reduction in EMG amplitude because we only tested at one muscle length where rFE might be independent of lengthening amplitude^29^, and we only powered the study to detect large effects (*d*_z_ of 1) with 80 to 90% power. We also did not use high-density EMG or estimate rFD, but assumed that rFD was present during fixed-end contractions based on previous experiments on the same muscle group within the same laboratory under similar conditions^16,17^. Further, we assumed that rFD would increase with increasing muscle activity (based on TA’s EMG amplitude) and active force production alongside additional fascicle shortening in line with previous animal findings^15,28^. However, it is possible that no additional rFD was induced in the initially-higher force contractions as rFD was found to be independent of shortening amplitude during submaximal (but not maximal^49^) voluntary contractions^17,50,51^. It is equally possible that lengthening-induced rFE precisely offset the magnitude of shortening-induced rFD, or that shortening-induced rFD simply decreased as some muscle fibers were decruited during a reduction in muscle activity (i.e. EMG amplitude) in the test conditions. Unfortunately though, we cannot provide experimental evidence for or against such interpretations. It is also possible that antagonistic muscles, such as the triceps surae, contributed to the recorded net joint torque and TA’s EMG signal through co-contraction and cross-talk, and it is a limitation that we did not record triceps surae muscle activities during the fixed-end dorsiflexion contractions. However, we think that systematic differences between conditions were unlikely (Supplementary Fig. 1) and negligible based on previous work^52,53^. Similarly, changes in synergistic activation between conditions were probably unlikely because we observed negligible fatigue during the contractions (Tables 1 and 2) and TA fascicle lengths were similar at similar torques and EMG amplitudes, except in Experiment 2 (where it is more likely that differences in fascicle length were due to fascicle tracking drift than to activation differences, which is a limitation of our utilized optical-flow-based tracking method).

Although active MTU lengthening can lead to indirect measures of rFE such as a subsequent reduction in steady-state EMG amplitude at a given net joint torque, fascicle lengthening at a fixed MTU length during a reduction in muscle activity based on TA’s EMG amplitude does not subsequently lower the EMG amplitude relative to torque-matched reference contractions. Consequently, contraction history effects during fixed-end contractions are limited to rFD, and the mechanical effects induced during fascicle lengthening and EMG amplitude reduction are probably not worth accounting for in muscle models that predict muscle force. On the contrary, the neural mechanisms contributing to reduced torque steadiness following large fascicle lengthening amplitudes and EMG amplitude reductions should be explored further.

## Supplementary Information

Below is the link to the electronic supplementary material.

Supplementary Material 1

## Data Availability

Datasets generated and analyzed during the current study are available in the following Zenodo repository [https://doi.org/10.5281/zenodo.19376065].
