# Supplementary material for: Fascicle lengthening during a large torque reduction subsequently decreases dorsiflexion torque steadiness
Source: Sci Rep. 2026 May 26;16:16285. doi: 10.1038/s41598-026-52001-z (PMC13212715; doi:10.1038/s41598-026-52001-z)
Supplement: Supplementary file 1 — Supplementary Material 1 [file 41598_2026_52001_MOESM1_ESM.docx]

**
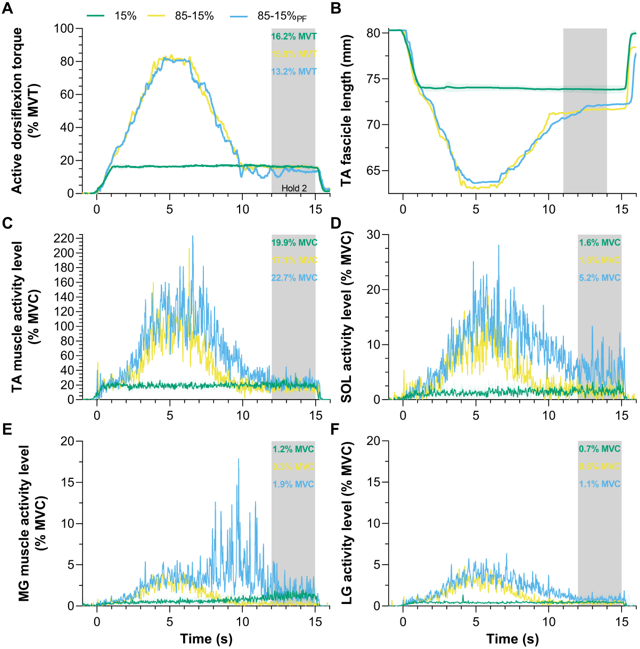
Supplementary Figure 1.** (A) Normalized active torque-time, (B) tibialis anterior (TA) muscle fascicle length change-time, (C) normalized TA EMG amplitude-time, and normalized soleus (D: SOL), medial gastrocnemius (E: MG), and lateral gastrocnemius (F: LG) EMG amplitude-time traces for single trials of one participant in Experiment 2. Only one of the three reference conditions (15% MVT) and one of the three test conditions (85-15% MVT) are shown. An additional condition, the 85-15%_PF_ MVT condition, was also performed here to show how the results are affected by intentional co-contraction; in this condition, the participant intentionally co-contracted their plantar flexors during and after the descending ramp (_PF_). Although five trials of the 85-15%_PF_ MVT condition were performed, all had a torque-matching variability that exceeded the 10% MVT required to be valid in Experiment 2, so only the trial with the best torque-matching (11.0% MVT) is shown here, alongside trials from the 15% MVT (3.2%) and 85-15% MVT (9.2%) conditions. The grey shaded area indicates the analyzed steady-state phase (12-15 s) of the contractions, which was delayed 1 s compared with the actual analysis because of the difficulty of matching torque while intentionally co-contracting. Mean values for normalized active torque (A) and EMG amplitude (C-F) during Hold 2 in each condition are shown. These values indicate that relative to the reference condition, increased co-contraction in the 85-15%_PF_ condition of 0.4 to 3.6% MVC decreased dorsiflexion torque by -3% MVT, despite a higher TA EMG amplitude of 2.8% MVC. As we did not observe such findings in the 85-15% MVT condition here (mean difference relative to 15% MVT in co-contraction: -0.9 to 0.2% MVC; torque: -0.3% MVT; and TA EMG amplitude: -2.8% MVC) or in either experiment, it is unlikely that plantar flexion co-contraction was substantially different in our test versus reference contractions.
